# The Role of PPAR**γ** in the Cyclooxygenase Pathway in Lung Cancer

**DOI:** 10.1155/2008/790568

**Published:** 2008-08-27

**Authors:** Saswati Hazra, Katherine A. Peebles, Sherven Sharma, Jenny T. Mao, Steven M. Dubinett

**Affiliations:** UCLA Lung Cancer Research Program, Division of Pulmonary and Critical Care Medicine and Hospitalists, Department of Medicine, Jonsson Comprehensive Cancer Center, David Geffen School of Medicine at UCLA, Los Angeles, CA 90095, USA

## Abstract

Decreased expression of peroxisome proliferator activated receptor-*γ* (PPAR*γ*) and high levels of the proinflammatory enzyme cyclooxygenase-2 (COX-2) have been observed in many tumor types. Both reduced (PPAR*γ*) expression and elevated COX-2 within the tumor are associated with poor prognosis in lung cancer patients, and recent work has indicated that these signaling pathways may be interrelated. Synthetic (PPAR*γ*) agonists such as the thiazolidinedione (TZD) class of anti-diabetic drugs can decrease COX-2 levels, inhibit growth of non-small-cell lung cancer (NSCLC) cells in vitro, and block tumor progression in xenograft models. TZDs alter the expression of COX-2 and consequent production of the protumorigenic inflammatory molecule prostaglandin E2 (PGE2) through both (PPAR*γ*) dependent and independent mechanisms. Certain TZDs also reduce expression of PGE2 receptors or upregulate the PGE2 catabolic enzyme 15-prostaglandin dehydrogenase. As several COX-2 enzymatic products have antitumor properties and specific COX-2 inhibition has been associated with increased risk of adverse cardiac events, directly reducing the effects or concentration of PGE2 may provide a more safe and effective strategy for lung cancer treatment. Understanding the mechanisms underlying these effects may be helpful for designing anticancer therapies. This article summarizes recent research on the relationship between (PPAR*γ*), TZDs, and the COX-2/PGE2 pathways in lung cancer.

 Despite the many advances made in diagnostic and treatment strategies, lung cancer remains the leading cause of cancer-related mortality in the United States and is responsible for more deaths than prostate, colon, and breast cancers combined [1]. Investigating the molecular mechanisms underlying the pathogenesis of lung cancer provides opportunities to develop innovative therapies that may reduce suffering due to this devastating disease. Decreased expression of peroxisome proliferator activated receptor (PPAR*γ*) originally identified as a regulator of glucose metabolism and adipocyte differentiation [2] has been associated with poor prognosis in lung cancer patients [3]. PPAR*γ* affects inflammatory gene expression, cell division, apoptosis, invasion, release of proangiogenic cytokines, and differentiation in many cancer types including lung cancer [4–8]. These properties have prompted extensive research on PPAR*γ* in cancer treatment and prevention. Members of the thiazolidinedione (TZD) class of PPAR*γ* agonists are currently approved for treatment of diabetes, and elicit many of the antitumor properties of PPAR*γ* activation through both PPAR*γ* dependent and independent pathways [9–14]. Several studies have demonstrated elevated constitutive expression of the inducible proinflammatory enzyme, cyclooxygenase-2 (COX-2) in human lung cancer [15–19]. Mounting evidence from investigations into the molecular effects of COX-2 over-expression in lung tumor cells indicates that this enzyme has a multifaceted role in conferring the malignant and metastatic phenotypes. The COX-2 enzymatic product prostaglandin E2 (PGE2) has been implicated in apoptosis resistance [20–22], angiogenesis [23, 24], decreased host immunity [25, 26], and enhanced invasion and metastasis [27–29]. This review summarizes investigations in the relationship between PPAR*γ*, its ligands, and COX-2 and PGE_2_ in lung cancer.

The PPAR family consists of three isoforms: PPAR*α*, PPAR*γ*, and PPAR*δ*, each encoded by different genes. PPARs are members of the nuclear hormone class of receptors and are involved in energy metabolism through transcriptional regulation of specific gene sets. Observations regarding high PPAR*γ* expression in adipose tissue in combination with its role in lipid and glucose homeostasis led to the development of the TZD class of PPAR*γ* agonists, including troglitazone, ciglitazone, rosiglitazone, and pioglitazone as antidiabetic and insulin-sensitizing drugs. Rosiglitazone and pioglitazone are currently approved for treatment of type 2 diabetes mellitus [30], and this class of drugs has been clinically available for approximately a decade. Some of the TZDs have been shown to exert anti-inflammatory [31], antiproliferative [32], and antiangiogenic effects [4]. The COX metabolite 15d-PGJ_2_ is a natural PPAR*γ* ligand and is considered a negative regulator of inflammatory and immune responses [33]. More recent results indicating that PPAR*γ* activation may attenuate inflammatory responses and cancer progression have led to extensive investigation into the role of this protein in inflammation and carcinogenesis.

PPAR*γ* is expressed in human non-small-cell lung cancer (NSCLC) and small cell lung carcinoma [34], and the expression of PPAR*γ* has been correlated with tumor histological type and grade [35]. In NSCLC, decreased PPAR*γ* expression was correlated with poor prognosis [3]. TZDs inhibit tumor formation in a variety of animal models, including colon [36] and lung cancers [37], and PPAR*γ* over-expression protects against tumor development in a mouse model of lung tumorigenesis [38]. Further, increased PPAR*γ* activity promotes epithelial differentiation of NSCLC cells in 3D culture [5]. It has also been shown that PPAR*γ* inhibits the growth of NSCLC in vitro and in vivo [5, 39, 40].

Cyclooxygenase is the rate-limiting enzyme for production of prostaglandins and thromboxanes from free arachidonic acid [41, 42]. Two COX isoforms, COX-1 and COX-2, have been extensively studied. COX-1 is constitutively expressed in most cells and tissues. COX-2 is an inducible enzyme that acts to produce prostaglandins and/or thromboxanes during an acute inflammatory response. The direct enzymatic product of COX-2 and PGH_2_ is converted to prostaglandins or thromboxanes by individual isomerases or prostaglandin synthases, and relative production of the various COX-2 products depends upon cellular concentrations of down-stream metabolic and catabolic enzymes within the COX-2 pathway. In NSCLC, the major eicosanoid produced is prostaglandin E2 (PGE_2_) through microsomal PGE_2_ synthase (mPGES) activity. The nicotinamide adenine dinucleotide positive-dependent catabolic enzyme 15-hydroxyprostaglandin dehydrogenase (15-PGDH) metabolizes PGE_2_ to biologically inactive 15-keto derivatives. The final PGE2 concentration experienced by NSCLC cells depends upon expression of PGES and 15-PGDH. A large body of evidence indicates that increased PGE2 production contributes to tumorigenesis. COX-2 over-expression is frequently observed in NSCLC, and the accompanying increased proliferation, invasion, angiogenesis, and resistance to apoptosis have been attributed in part to elevated PGE_2_ production in the vicinity of the tumor. Thus, COX-2 and its downstream signaling pathways represent potential targets for lung cancer chemoprevention and therapy.

Studies indicate that COX-2 and PPAR*γ* signaling pathways are intertwined. PPAR*γ* ligands suppress COX-2 expression induced by LPS and PMA in macrophages, astrocytes, and epithelial cells [43–45]. The COX-2 metabolite 15d-PGJ2 is an endogenous ligand for PPAR*γ* [46], and during resolution of inflammation elevated 15d-PGJ2 production downregulates COX-2 through a negative feedback loop involving PPAR*γ* and NF-*κ*B [44, 47]. Synthetic and endogenous PPAR*γ* ligands decrease the high COX-2 expression associated with several malignancies including cervical [48] and liver cancers [49] and forced PPAR over-expression decreases COX-2 levels in lung cancer cells [38]. While PPAR*γ* agonists decrease COX-2 expression or prevent COX-2 induction in most settings, COX-2 expression is increased in some studies [50, 51]. For example, Ikawa et al. reported that rosiglitazone (also known as BRL49653) increases COX-2 expression in human colorectal carcinoma cells [52]. PPAR*γ* ligands also have been shown to induce COX-2 expression in mammary epithelial cells [53], monocytes [54], and human synovial fibroblasts [55]. The effect of PPAR*γ* agonists on COX-2 expression may vary based upon the cell type, the specific agonist molecule, and the presence of additional inflammatory mediators. Off-target effects of TZDs may also partially account for differences in the effects of these molecules on COX-2 expression.

Although TZDs are widely known as ligands for PPAR*γ*, they may mediate receptor-independent effects, as previously reported [56–58]. For example, by using embryonic stem cells from PPAR*γ*-null mice, Chawla et al. found that neither macrophage differentiation nor anti-inflammatory effects of synthetic PPAR*γ* ligands are PPAR*γ* receptor-dependent. To distinguish the effects of PPAR*γ* from off-target effects of PPAR*γ* ligands in lung cancer cells, Bren-Mattison et al. utilized a molecular approach to over-express PPAR*γ* in two NSCLC cell lines and assessed the direct effect of PPAR*γ*. Their goal was to determine whether the antitumorigenic effects of PPAR*γ* were mediated via COX-2 pathways in NSCLC. Their results clearly demonstrated that exogenously expressed PPAR*γ* suppresses COX-2 promoter activity and protein expression resulting in suppression of PGE_2_ production [38]. The COX-2 promoter has binding sites for cAMP response element, NF-IL-6, and NF-*κ*B [59]. Although the COX-2 promoter contains multiple regulatory elements, their data indicate that the inhibitory effects of PPAR*γ* are mediated through NF-*κ*B. Several studies have demonstrated that constitutive activation of NF-*κ*B in tumor cells is mediated through activation of Akt [60, 61]. Bren-Mattison et al. demonstrated that the inhibitory effects of PPAR*γ* on COX-2 were mediated via increased activity of PTEN leading to decreased phospho-Akt and inhibition of NF-*κ* *β* [38]. These authors further demonstrated that transgenic mice over-expressing PPAR*γ* exhibited reduced COX-2 in type II alveolar epithelial cells of lung, and those mice were protected against lung cancer development in a chemical carcinogenesis mouse model [38]. In summary, these data indicate that COX-2 downregulation may mediate some of the antitumorigenic effects of PPAR*γ* over-expression.

The PPAR*γ* agonists may also affect COX-2 in a PPAR*γ* independent manner (see Table 1). For example, in A549 NSCLC cells troglitazone enhanced both COX-2 and mPGES expression in a concentration dependent manner, resulting in a marked increase in PGE_2_ production [62]. Cotreatment with the PPAR*γ* antagonists GW9662 and bisphenol A diglycidyl ether (BADGE) had no effect on COX-2 induction by troglitazone indicating that this event is PPAR*γ* independent. Troglitazone increased COX-2 expression at least in part by activating ERK and PI3K pathways, which have been previously demonstrated to influence COX-2 levels [63–65]. Combined troglitazone and TNF*α* stimulation resulted in additive enhancement of COX-2 expression. The COX-2 metabolite 15d-PGJ_2_ had no detectable effects on COX-2 or mPGES expression or PGE_2_ production in A549 cells. This is consistent with the hypothesis that PPAR*γ*-independent mechanisms can partially account for discrepancies in the effects of different TZD drugs on COX-2 expression. Thus, in lung cancer, PPAR*γ* agonists appear to regulate COX-2 expression and affiliated protumorigenic cellular phenotypes through both PPAR*γ* dependent and independent means.

We recently examined the effect of the pioglitazone and rosiglitazone on COX-2 and PGE_2_ levels in A427 and A549 NSCLC cells. Both TZDs inhibited PGE_2_ production in NSCLC cells via a COX-2 independent pathway. To define the mechanism underlying COX-2 independent suppression of PGE_2_ production, we focused on the prostaglandin synthases that are responsible for the PGE_2_ production and on 15PGDH the catabolic enzyme responsible for its degradation to biologically inactive15-keto derivatives [66]. None of the three prostaglandin synthases (microsomal PGES1, PGES2, and cytosolic PGES) was downregulated by pioglitazone or rosiglitazone, however, 15-PGDH was induced by TZDs. TZD-mediated suppression of PGE_2_ concentration was significantly inhibited by small interfering RNA to 15-PGDH. Studies using dominant-negative PPAR*γ* over-expression or GW9662 revealed that the induction of 15-PGDH by both pioglitazone and rosiglitazone is PPAR*γ*-independent.These findings indicate that it is possible to use a clinically available pharmacological intervention to suppress tumor-derived PGE_2_ by enhancing catabolism rather than blocking synthesis. The potential benefits of inhibiting PGE_2_ levels in a COX-2-independent manner include the following. First, promoting 15-PGDH activity could decrease PGE_2_ without modifying other prostaglandins such as PGI_2_. This is potentially important because the latter has been noted to have antitumor properties [67]. It has been suggested that a ratio of PGs may be important in regulating the malignant phenotype. Thus, inhibiting COX-2 activity would diminish both PGE_2_ and PGI_2_, whereas selective induction of 15-PGDH could lead to a more favorable PGI_2_/PGE_2_ ratio. Second, suppression of PGE_2_ levels without alteration in COX-2 may limit some of the cardiovascular toxicities associated with COX-2 inhibition [68]. Finally, unlike COX-2 inhibition, which may lead to upregulation of certain leukotrienes that favor malignant progression [69], 15-PGDH induction may lead only to a decrement of PGE_2_. This speculation will require further investigation.

Different TZDs have the capacity to modulate arachidonic acid metabolism by a variety of pathways (see Figure 1). Recent evidence indicates that ciglitazone induces differentiation and apoptosis in NSCLC [7]. The mechanisms of ciglitazone's capacity to modulate PGE2 levels in lung adenocarcinoma cells were recently reported [13]. In contrast to pioglitazone and rosiglitazone, ciglitazone mediates COX-2 dependent suppression of PGE_2_ in NSCLC. Ciglitazone treatment suppressed COX-2 mRNA expression and COX-2 promoter activity but did not modify the expression of enzymes downstream of COX-2 including PGES and 15-PGDH. Utilization of dominant-negative PPAR*γ* showed that suppression of COX-2 and PGE2 by ciglitazone is mediated via non-PPAR pathways.

PPAR*γ* ligands may also interfere with protumorigenic signals derived from COX-2 by interrupting the function of PGE_2_ G-protein coupled receptors (GPCRs) designated E-prostanoid (EP) receptors 1–4 [70]. Han and Roman found that in NSCLC cell lines, the PPAR*γ* ligands GW1929, 15 dPGJ_2_, ciglitazone, troglitazone, and rosiglitazone significantly decreased EP2 mRNA and protein levels causing growth inhibition in NSCLC cells [71]. The inhibitory effects of rosiglitazone and ciglitazone but not 15d-PGJ2 were suppressed by the PPAR*γ* antagonist GW9662 suggesting the involvement of PPAR*γ*-dependent and independent mechanisms.

Recently, a retrospective study by Govindarajan et al. demonstrated a significant reduction in lung cancer risk in diabetic patients using the TZD rosiglitazone [72]. Importantly, several clinical studies in diabetes patients have demonstrated an increased risk of cardiovascular events associated with rosiglitazone or pioglitazone treatment [73–75]. This is of particular significance in light of cardiovascular toxicity associated with COX-2 inhibition. Recently, several chemoprevention trials are being initiated using TZDs [76]. However, adverse cardiac events are associated with chronic TZD treatment [74]. Based on these findings, future clinical studies attempting to utilize TZDs in prevention of cancer will require selection of patient populations without cardiovascular risk. Prospective clinical studies specifically designed to address the effects of TZDs on cancer, and cardiac outcomes are required. If the anti-inflammatory and antitumor effects of TZDs are derived through pathways distinct from those leading to cardiovascular toxicity, more selective candidate drug molecules may be therapeutically effective, without leading to adverse cardiac events. Thus, more research is required to define opportunities to specifically interfere with PGE_2_ production, metabolism, or downstream effects. This could ultimately lead to reduction in lung cancer growth or prevention while leaving the steady-state concentrations of desirable eicosanoids in tact [77].

Both elevated COX-2 and reduced PPAR*γ* expression are associated with poor prognosis in lung cancer patients [3, 78–80] and recent work has revealed multiple interactions between PPAR*γ* signaling and the COX-2 pathway. The COX-2 product 15d-PGJ_2_ is an endogenous ligand for PPAR*γ*, and PPAR*γ* activation as a result of elevated 15d-PGJ_2_ results in COX-2 downregulation in an autoregulatory feedback loop that may contribute to natural resolution of the inflammatory response [46]. Forced expression of PPAR*γ* decreases COX-2 expression in cultured human NSCLC cells and mouse lungs and protects against lung tumor development in a murine model [5, 38]. Synthetic PPAR*γ* ligands, several of which are currently approved for treatment of diabetes, can interrupt several stages of the COX-2/PGE_2_ protumorigenic pathway, although in certain cases PPAR*γ* ligands may increase COX-2 expression. These effects are primarily mediated through PPAR*γ*-independent pathways (see Table 1). PPAR*γ* ligands may directly decrease COX-2 transcription in an NF-*κ*B-dependent manner [38], or they can interfere with downstream targets such as the PGE_2_ receptor EP2 [71] or the enzyme responsible for PGE2 catabolism, 15-PGDH [66]. The targets downstream of COX-2 may be useful in light of recent evidence that interfering with COX-2 enzymatic activity may increase risk of cardiovascular events [68]. The discovery that certain PPAR*γ* agonists can specifically reduce PGE_2_ concentration or expression of EP receptors may aid in the design of strategies to reduce the effects of harmful prostaglandins without impacting production of critical cardioprotective eicosanoids.

## Figures and Tables

**Figure 1:**
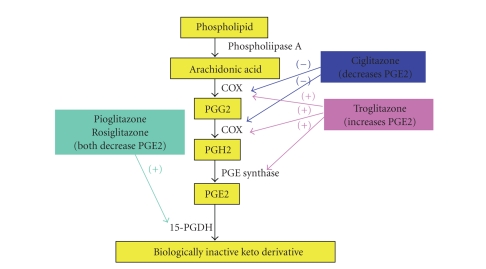
Effects of various TZDs on the PGE2 pathway.

**Table 1:** Off-target effects of TZDs in NSCLC.

Thiazolidinediones	Molecular effects	Mechanisms	Reference
Troglitazone	↑ PGE2	↑ COX-2, ERK and PI3K phosphorylation	[62]
Pioglitazone, Rosiglitazone	↓ PGE2	↑ 15-PGDH	[14]
Ciglitazone	↓ PGE2	↓ COX-2	[13]
